# Jejunal Insulin Signalling Is Increased in Morbidly Obese Subjects with High Insulin Resistance and Is Regulated by Insulin and Leptin

**DOI:** 10.3390/jcm9010196

**Published:** 2020-01-10

**Authors:** Carolina Gutierrez-Repiso, Ailec Ho-Plagaro, Concepción Santiago-Fernandez, Sara Garcia-Serrano, Francisca Rodríguez-Pacheco, Sergio Valdes, Lourdes Garrido-Sanchez, Cristina Rodríguez-Díaz, Carlos López-Gómez, Francisco J. Moreno-Ruiz, Guillermo Alcain-Martinez, Amandine Gautier-Stein, Gilles Mithieux, Eduardo Garcia-Fuentes

**Affiliations:** 1 Instituto de Investigación Biomédica de Málaga-IBIMA, 29009 Málaga, Spain; 2 Unidad de Gestión Clínica de Endocrinología y Nutrición, Hospital Universitario Virgen de la Victoria, 29009 Málaga, Spain; 3 Unidad de Gestión Clínica de Aparato Digestivo, Hospital Universitario Virgen de la Victoria, 29009 Málaga, Spain; 4 Facultad de Ciencias, Universidad de Málaga, 29009 Málaga, Spain; 5 Facultad de Medicina, Universidad de Málaga, 29009 Málaga, Spain; 6 Unidad de Gestión Clínica de Endocrinología y Nutrición, Hospital Regional Universitario, 29009 Málaga, Spain; 7 CIBER de Diabetes y Enfermedades Metabólicas Asociadas-CIBERDEM, 29009 Málaga, Spain; 8 CIBER Fisiopatología de la Obesidad y Nutrición-CIBEROBN, 29009 Málaga, Spain; 9 Unidad de Gestión Clínica de Cirugía General, Digestiva y Trasplantes, Hospital Regional Universitario, 29009 Málaga, Spain; 10 Institut National de la Santé et de la Recherche Médicale, U1213, F-69008 Lyon, France; 11 Université de Lyon, F-69008 Lyon, France; 12 Faculté Lyon-Est Laennec, Université de Lyon 1, F-69622 Villeurbanne, France

**Keywords:** insulin resistance, jejunum, morbid obesity, leptin

## Abstract

Little is known about the jejunal insulin signalling pathways in insulin resistance/diabetes states and their possible regulation by insulin/leptin. We study in jejunum the relation between insulin signalling and insulin resistance in morbidly obese subjects with low (MO-low-IR) or with high insulin resistance (MO-high-IR), and with type 2 diabetes treated with metformin (MO-metf-T2DM), and the effect of insulin/leptin on intestinal epithelial cells (IEC). Insulin receptor substrate-1 (IRS1) and the catalytic p110β subunit (p110β) of phosphatidylinositol 3-kinase (PI3K) were higher in MO-high-IR than in MO-low-IR. The regulatory p85α subunit of PI3K (p85α)/p110β ratio was lower in MO-high-IR and MO-metf-T2DM than in MO-low-IR. Akt-phosphorylation in Ser473 was reduced in MO-high-IR compared with MO-low-IR. IRS1 and p110-β were associated with insulin and leptin levels. The improvement of body mass index (BMI) and HOMA-IR (homeostasis model assessment of insulin resistance index) after bariatric surgery was associated with a higher IRS1 and a lower p85α/p110β ratio. IEC (intestinal epithelial cells) incubation with a high glucose + insulin dose produced an increase of p85α and p110β. High dose of leptin produced an increase of IRS1, p85α and p110β. In conclusion, despite the existence of insulin resistance, the jejunal expression of genes involved in insulin signalling was increased in MO-high-IR. Their expressions were regulated mainly by leptin. IRS1 and p85α/p110β ratio was associated with the evolution of insulin resistance after bariatric surgery.

## 1. Introduction

Intestine may play an important role in glucose homeostasis [1,2,3]. Previous studies have shown the involvement of insulin resistance in the intestinal metabolism dysregulation [4]. However, changes in the human intestinal metabolism in insulin resistance and/or type 2 diabetes mellitus (T2DM) have not been explored in depth [3,5,6,7]. Intestinal glucose-6 phosphatase (G6Pase) and phosphoenolpyruvate carboxykinase—cytosolic form (PEPCK-c), which are involved in the gluconeogenesis pathway and in the control of endogenous glucose production [8], are targets of insulin [9]. They could be associated with insulin resistance and its evolution after bariatric surgery, on the other hand [1,10,11]. Moreover, the glucose entry into the enterocyte could be modulated through the regulation of glucose transporter 1 (GLUT1), a constitutive glucose transporter that is insulin-independent, and through the regulation of enzymes involved in gluconeogenesis/glycolysis. Hexokinase 1 (HK1) and L-type pyruvate kinase (PKLR) are the two main enzymes that regulate the glycolysis pathway [12,13]. These pathways could play an important role in the remission of T2DM after bariatric surgery.

Insulin sensitivity is mediated by insulin receptor substrate-1 (IRS1) and phosphatidylinositol 3-kinase (PI3K). A reduction in IRS protein expression has been found in obese, insulin resistant and diabetic patients in skeletal muscle and adipocytes [14,15]. On the other hand, an adequate balance between regulatory p85 and the catalytic p110 subunits of PI3K is crucial for the regulation of insulin signalling. Also, p85 could have a negative role as mediators of PI3K activation [16]. An excess of p85 monomers, not associated with p110, compete with the p85/p110 dimer and produce ineffective insulin signalling [17]. But, to date, the association between the intestinal insulin signalling pathways and the remission of obesity and T2DM after bariatric surgery has not been explored. Also, the influence of metformin, a drug that positively interferes with insulin sensitivity and intestinal glucose metabolism [18], is not fully known.

Insulin resistance has been widely documented in muscle and adipose tissue [1,19]. However, there is little information about the differences in intestinal insulin signalling pathways between subjects with low and high insulin resistance. Its possible regulation by insulin and leptin, two hormones closely involved in the regulation of obesity and insulin resistance, has not been systemically explored. Some evidence suggests a crosstalk between insulin and leptin signalling in the IRS1-PI3K pathway [20]. Understanding the molecular mediators linking these pathways is of particular importance [20,21].

Therefore, the aim of the present study was (a) to analyse the jejunal expression of genes involved in insulin signalling and glucose metabolism pathway in morbidly obese subjects according to the insulin resistance grade and in those treated with metformin, (b) to analyse the association with the change of body mass index (BMI) and insulin resistance of those subjects after bariatric surgery, and (c) to study the effect of insulin, glucose and leptin on the expression of relevant genes.

## 2. Research Design and Methods

### 2.1. Subjects

The study was undertaken in 45 morbidly obese subjects who underwent Roux-en-Y gastric bypass (RYGB) at baseline and 1, 3, 6 and 12 months after RYGB [3,5,22] (Table 1). The subjects were classified in three groups: two groups according to the homeostasis model assessment of insulin resistance (HOMA-IR) level (with low HOMA-IR value (<4.7) (MO-low-IR), *n* = 15) and with high HOMA-IR value (>4.7) (MO-high-IR, *n* = 15) (both groups without treatment for T2DM) and a third group (*n* = 15) with T2DM who were only receiving metformin treatment (MO-metf-T2DM) [3,5,23]. Subjects were excluded if they had T2DM and were receiving insulin treatment or other oral hypoglycaemic medications, had cardiovascular disease, acute inflammatory or infectious disease. All the participants gave their informed consent, and the study was reviewed and approved by the Ethics and Research Committee of the Regional University Hospital, Málaga, Spain. Samples from subjects were processed and frozen immediately after their reception at the Regional University Hospital Biobank (Andalusian Public Health System Biobank).

### 2.2. Laboratory Measurements

Blood samples were collected after 10–12 h fasting at baseline and 1, 3, 6 and 12 months after RYGB. Serum was separated and immediately frozen at −80 °C until analysis. Serum biochemical parameters were measured in duplicate, as previously described [3,5,6]. Changes in the variables due to RYGB were expressed as percentages and were calculated as (variable_baseline_ − variable_1, 3, 6 or 12 months_) × 100/variable_baseline_ [24].

### 2.3. Jejunal Biopsy Samples

Jejunal biopsy samples (*n* = 15 per group) were obtained during bariatric surgery, 40 cm from the ligament of Treitz [3,5,6]. The mucosa was washed with physiological saline solution, scraped, immediately frozen in liquid nitrogen and maintained at −80 °C until analysis.

### 2.4. Cell Viability in Jejunum

A cell viability assay in jejunal biopsy samples (*n* = 6 per group) was performed in triplicate using the CellTiter-Glo^®^ Luminescent Cell Viability Assay (Promega, Southampton, UK) according to the manufacturer’s instructions [5].

### 2.5. Intestinal Epithelial Cells (IEC) Isolation and Incubation

For this experiment, jejunal biopsy samples were only obtained from MO-low-IR subjects (*n* = 6) during RYGB since this group was the one that had less metabolic alterations and could be considered as the control group of the three groups of subjects studied. IEC were isolated as previously described [3,5]. Isolated IEC were cultured in 24-well plates (200,000 cells/well) with DMEM supplemented with 1% fetal bovine serum, 1% l-glutamine, 1% penicillin and streptomycin at 37 °C and 5% CO_2_ for 3 h. Tests were performed in different conditions: 5.5 mM glucose, 5.5 mM glucose + 100 nM insulin, 25 mM glucose, 25 mM glucose + 100 nM insulin, leptin 50 mg/mL and leptin 150 mg/mL. Each treatment was performed in triplicate. 

### 2.6. Western Blot

Jejunal proteins (*n* = 3 per group) were extracted with RIPA buffer (AMRESCO, Inc., Solon, OH, USA) and protease and phosphatase inhibitor cocktails (Sigma-Aldrich, St. Louis, MO, USA). Homogenates were centrifuged at 14,000× *g* rpm for 10 min at 4 °C, and supernatants were aliquoted and immediately frozen at −80 °C until analysis. Protein levels were analysed using the bicinchoninic acid (BCA) method (Thermo Fisher Scientific Inc., Rockford, IL, USA). Proteins (50 mg) were denatured in 0.125 M Tris-HCl (pH 6.8), 20% glycerol, 4% SDS and 10% β-mercaptoethanol, and subjected to SDS-PAGE on polyacrylamide 4%–20% Mini-PROTEAN^®^ TGX™ Precast Protein Gels (Bio-Rad, Hercules, CA, USA) and electrotransferred on a polyvinylidene fluoride membrane (Trans-Blot^®^ Turbo™ Midi PVDF) (Bio-Rad, Hercules, CA, USA). Membranes were blocked in TBS-Tween-20 (50 mmol/L Tris-HCl (pH 7.5), 0.15 mol/L NaCl and 0.1% Tween-20) containing 5% skimmed milk for 1 h at room temperature. Afterwards, membranes were incubated with phospho-Akt serine/threonine kinase 1 (phospho-Akt) (Ser473) monoclonal antibody at 1:1000 dilution (44-621G, ThermoFisher Scientific Inc., Rockford, IL, USA) and human/mouse/rat Akt pan specific antibody at 0.2 µg/mL (MAB2055, R&D Systems, Inc., Minneapolis, MN, USA) overnight at 4 °C. Membranes were washed (3 × 5 min, 50 mmol/L Tris-HCl (pH 7.5), 0.15 mol/L NaCl and 0.1% Tween-20) and incubated with VeriBlot for IP Detection Reagent (HRP) (ab131366, Abcam plc, Cambridge, UK) at 1:4000 for 2 h at room temperature. We used Clarity Max™ Western ECL Blotting Substrates (Bio-Rad, Hercules, CA, USA) to easily and quickly visualize proteins with shorter acquisition times. Proteins were visualized using ImageQuant LAS 4000 (GE Healthcare Bio-Sciences AB, Uppsala, Sweden), and quantified with the image acquisition analysis software ImageJ (National Institute of Mental Health, Bethesda, MD, USA).

### 2.7. RNA Extraction and RT-PCR

Total RNA from the jejunal biopsy samples (*n* = 45) and IEC incubations were obtained using RNeasy Mini Kit (Qiagen GmbH, Stockach, Germany) [3,5,25]. cDNA was synthesized by retrotranscription using the M-MLV retrotranscriptase (Sigma-Aldrich, St. Louis, MO, USA). Gene expression levels were analysed in triplicate by quantitative real-time reverse transcriptase polymerase chain reaction (RT-PCR) using ABI 7500 Fast Real-Time PCR System (Applied Biosystems, Foster City, CA, USA). 

Primers for the PCR reaction were designed based on NCBI database sequences and obtained from Proligo (Sigma-Aldrich, St. Louis, MO, USA):

18S (NM_022551, forward: gtcaatgtctgctttcctcaac, reverse: gttccagcatattttgcgagt), 

IRS1 (NM_005544.2, forward: caagaccatcagcttcgtga, reverse: agagtcatccacctgcatcc), 

p85α (NM_181524.1, forward: accccagtttttgttgcttg, reverse: actgcccaacaaaaccgtcc), 

p110β (NM_001256045.1, forward: ggatgttgccttatggctgt, reverse: ccttggagatgctgaaaagc), 

G6Pase (NM_001270397.1, forward: ctggagaaagcccagaggtg, reverse: aggacgagggaggctacaat), 

HK1 (NM_033498, forward: tcccaacaatgagtccaacc, reverse: gccacgatgtagtcaccttac), 

PKLR (NM_181871 and NM_000298, forward: atcatgctgtcaggggagac,

reverse: agtgggatcacggcttagtg),

GLUT1 (NM_006516, forward: ttcactgtcgtgtcgctgtt, reverse: ggccacgatgctcagatagg),

PEPCK (NM_002591, forward: gagagaactccagggtgctg, reverse: ccttggagatgctgaaaagc). 

Relative expression was calculated using the comparative Ct method (2^−Δ*C*t^), and expression results are given as the expression ratio relative to the 18S gene expression.

### 2.8. Enzymatic Activity of G6Pase

Enzymatic activity of G6Pase was analysed by a procedure described by Sigma-Aldrich [26] (Sigma-Aldrich, St. Louis, MO, USA). Briefly, a 10 µL aliquot of the tissue homogenate (*n* = 5 per group) was incubated with 100 mM BIS-TRIS buffer, pH 6.5, and 200 mM glucose-6-phosphate at 37 °C for 5 min, and afterwards, 20% trichloroacetic acid was added. Samples were mixed, incubated for 5 min at 25 °C and centrifuged at 4000 rpm for 10 min. The supernatant was incubated at 25 °C for 5 min for the colour development step with the Taussky–Shorr colour reagent, and absorbance readings at 660 nm were obtained. G6Pase was analysed in triplicate. Blanks for each sample and phosphorus standard curve were included. Results were adjusted by jejunal protein concentration, which was determined according to the bicinchoninic acid method (Thermo Fisher Scientific Inc. Rockford, IL, USA).

### 2.9. Statistical Analysis

All analyses were performed using R statistical software, version 2.8.1 (Department of Statistics, University of Auckland, Auckland, New Zealand; http://www.r-project.org/). The sample size was calculated based on a preliminary study. A one-way analysis of variance was used for mRNA expressions and G6Pase activity, and a repeated measurements analysis for incubated IEC, accepting an alpha risk of 0.05 and a beta risk of 0.2 in a two-sided test. Differences between groups were made with Kruskal–Wallis test. The statistical differences between before and after RYGB were made with Wilcoxon test [3,5]. Differences in the evolution of BMI and HOMA-IR (homeostasis model assessment of insulin resistance index) after RYGB, and between conditions of IEC incubations were made with a repeated measure ANOVA adjusted by Bonferroni test. Partial correlation coefficients (adjusted by BMI) were calculated to estimate the associations between variables. Multiple linear regression models were also used to determine the associations between variables. Values were considered to be statistically significant when the *p* ≤ 0.05. The results are given as the mean ± standard deviation (SD) or mean ± standard error of the mean (SEM) in figures.

## 3. Results

### 3.1. Jejunal Insulin Signalling

First, we analysed the cell viability in jejunal samples by the CellTiter-Glo^®^ Luminescent Cell Viability Assay. It was 97.2% ± 5.2% and 99.5% ± 6.2% in MO-high-IR and MO-metf-T2DM, respectively, when compared with MO-low-IR group (100%). No significant differences were found between groups.

**Figure 1:**
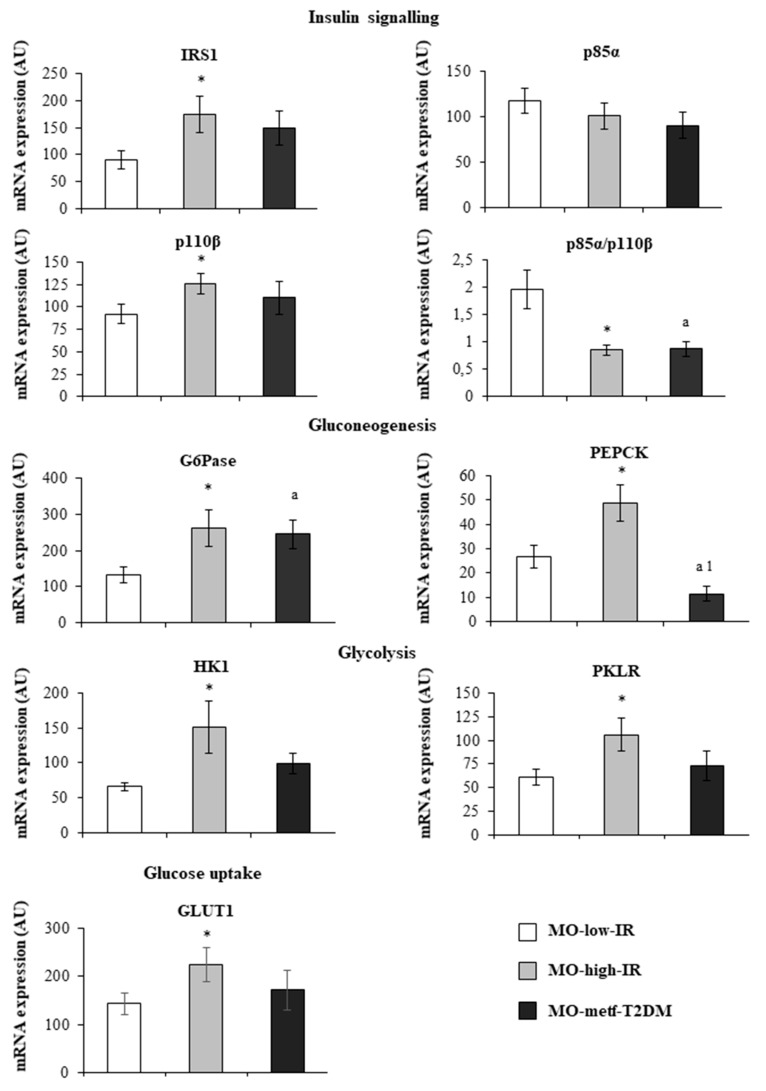
mRNA expression levels of studied genes in the jejunum of MO-low-IR (*n* = 15), MO-high-IR (*n* = 15) and in MO-metf-T2DM (*n* = 15). The results are given as mean ± SEM (standard error of the mean). * *p* < 0.05: significant differences with regard to MO-low-IR group. ^a^ *p* < 0.05: significant differences between MO-high-IR and MO-metf-T2DM groups. IRS1: insulin receptor substrate 1. p85α: phosphatidylinositol 3-kinase 85 kDa regulatory subunit alpha. p110β: phosphatidylinositol 3-kinase 110 kDa catalytic subunit beta. G6Pase: glucose-6 phosphatase. HK1: hexokinase 1. PKLR: L-type pyruvate kinase. GLUT1: solute carrier family 2 member 1 or glucose transporter 1. PEPCK: phosphoenolpyruvate carboxykinase. ^1^ *p* < 0.05.

In the MO-high-IR group, IRS1 (*p* = 0.037) and p110β (*p* = 0.041) expressions were higher than in the MO-low-IR group, while the p85α/p110β ratio was lower (*p* < 0.001) (Figure 1). Compared with the MO-low-IR, the p85α/p110β ratio (*p* = 0.010) was lower in the MO-metf-T2DM group (Figure 1).

**Figure 2:**
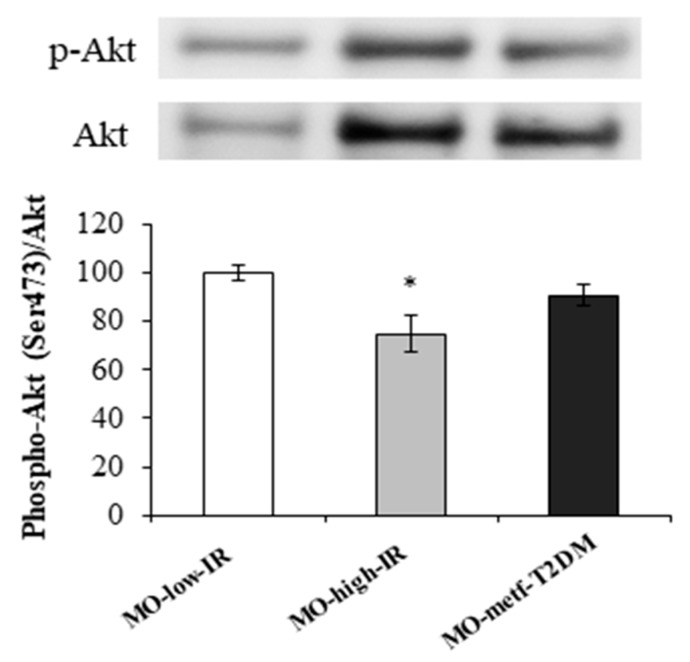
Akt phosphorylation state in jejunum of MO-low-IR (*n* = 3), MO-high-IR (*n* = 3) and MO-metf-T2DM (*n* = 3). Jejunal homogenates were analysed for the phosphorylation of Akt at Ser473. Densitometric analyses of phosphorylation were normalized for Akt. The results are given as mean ± SEM. * *p* < 0.05: significant differences with regard to MO-low-IR group.

We assessed phosphorylation of Akt, protein involved in the insulin signalling, in the jejunum of morbidly obese subjects. Phosphorylation of Akt in Ser473 was reduced in the jejunum of MO-high-IR (*p* = 0.005) (Figure 2) compared with MO-low-IR.

### 3.2. Jejunal Gluconeogenesis/Glycolysis

In the MO-high-IR group, the expression of gluconeogenic enzymes G6Pase (*p* = 0.024) and PEPCK (*p* = 0.027), the expression of glycolytic enzymes HK1 (*p* = 0.021) and PKLR (*p* = 0.048), and GLUT1 (*p* = 0.048) were higher than in the MO-low-IR group (Figure 1). Compared with the MO-low-IR group, the expression of the gluconeogenic enzyme PEPCK (*p* = 0.041) was lower and G6Pase (*p* = 0.014) was higher in the MO-metf-T2DM group (Figure 1).

**Figure 3:**
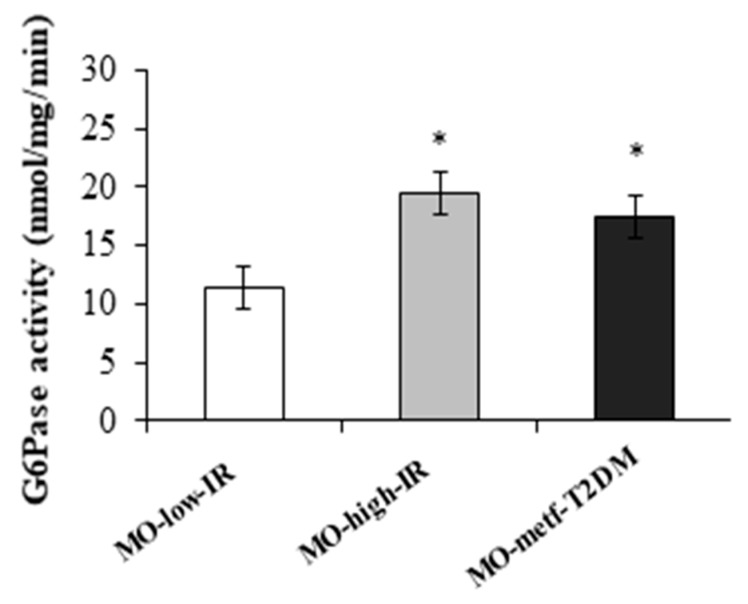
Enzymatic activity of G6Pase in the jejunum of MO-low-IR (*n* = 5), MO-high-IR (*n* = 5) and in MO-metf-T2DM (*n* = 5). The results are given as mean ± SEM. * *p* < 0.05: significant differences with regard to MO-low-IR group.

Also, the enzymatic activity of G6Pase was higher in MO-high-IR (*p* = 0.005) and MO-metf-T2DM (*p* = 0.020) groups than in MO-low-IR group (Figure 3).

### 3.3. Significant Associations between Jejunal mRNA Expression Levels and Biochemical and Anthropometric Baseline Variables

We studied these associations in all subjects together (*n* = 45). p85α/p110β ratio correlated negatively with IRS1 (*r* = −0.385, *p* = 0.042) and p110β (*r* = −0.547, *p* = 0.002) expressions. Also, we found different significant correlations between mRNA expression levels with anthropometric and biochemical variables, mainly insulin, HOMA-IR, BMI and waist circumference (Table 2). There were no other significant correlations.

In order to strengthen the independence of these associations as predictors of mRNA gene expression, multiple regression analysis models were constructed for each gene. The mRNA expression levels of most of the genes studied were associated with insulin or leptin levels (Table 3).

### 3.4. Associations between mRNA Gene Expression Levels and Changes in BMI and HOMA-IR after RYGB

BMI and HOMA-IR data presurgery and 1, 3, 6 and 12 months after RYGB are shown in Table 4. We found a significant improvement of these variables after RYGB. The improvement of BMI and HOMA-IR 1, 3, 6 and 12 months after RYGB was associated with the IRS1, HK1 and G6Pase expression, and p85α/p110β ratio (Table 5). No other significant correlations were found (data not shown).

### 3.5. Insulin Effects on the mRNA Expression Levels in Incubated IEC

**Figure 4:**
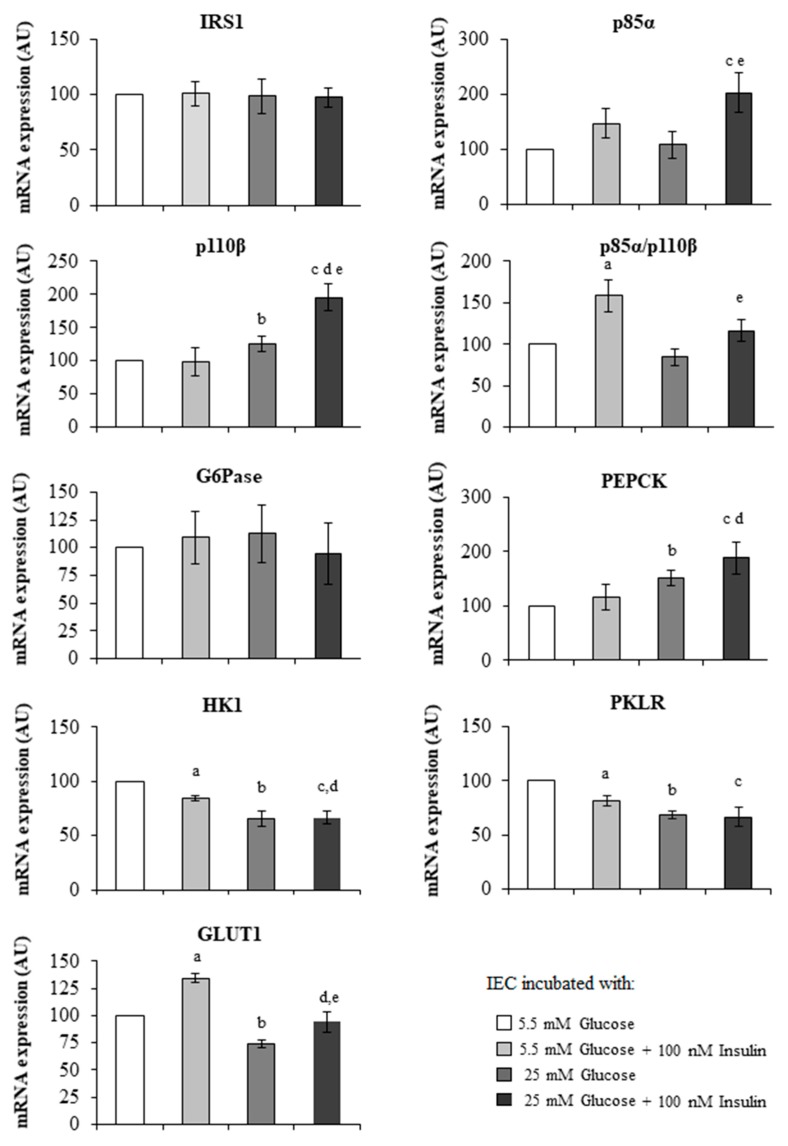
mRNA expression levels of studied genes in intestinal epithelial cells (IEC) incubations obtained from jejunum samples of MO-low-IR (*n* = 6). IEC were isolated and incubated as described in the Material and Methods section. IEC were incubated with 5.5 mM glucose, 5.5 mM glucose + 100 nM insulin, 25 mM glucose, and 25 mM glucose + 100 nM insulin. The results were expressed as a percentage of 5.5 mM glucose level (100%). ^a^ Significant difference between 5.5 mM glucose and 5.5 mM glucose + 100 nM insulin (*p* < 0.05). ^b^ Significant differences between 5.5 mM glucose and 25 mM glucose (*p* < 0.05). ^c^ Significant differences between 5.5 mM glucose and 25 mM glucose + 100 nM insulin (*p* < 0.05). ^d^ Significant differences between 5.5 mM glucose + 100 nM insulin and 25 mM glucose + 100 nM insulin (*p* < 0.05). ^e^ Significant differences between 25 mM glucose and 25 mM glucose + 100 nM insulin (*p* < 0.05). The results were given as the mean ± SEM. IRS1: insulin receptor substrate 1. p85α: phosphatidylinositol 3-kinase 85 kDa regulatory subunit alpha. p110β: phosphatidylinositol 3-kinase 110 kDa catalytic subunit beta. G6Pase: glucose-6 phosphatase. HK1: hexokinase 1. PKLR: L-type pyruvate kinase. GLUT1: solute carrier family 2 member 1 or glucose transporter 1. PEPCK: phosphoenolpyruvate carboxykinase.

Given the significant associations found between insulin and mRNA gene expression levels, we checked whether insulin could modify the gene expression of IEC (Figure 4).

#### 3.5.1. Jejunal Insulin Signalling

Regarding the IEC treated with 5.5 mM glucose (Figure 4), the incubation with a high dose of insulin (5.5 mM glucose + 100 nM insulin) produced an increase of the p85α/p110β ratio (*p* = 0.021). The incubation with high levels of glucose (25 mM glucose) produced an increase of p110β (*p* = 0.046).

In the IEC incubated with a high dose of glucose and insulin (25 mM glucose + 100 nM insulin) (Figure 4), p110β expression (*p* = 0.018) was higher than in the IEC incubated with a high dose of insulin (5.5 mM glucose + 100 nM insulin). p85α (*p* = 0.018) and p110β (*p* = 0.028) expressions were higher and the p85α/p110β ratio (*p* = 0.043) lower than in the IEC incubated with a high dose of glucose (25 mM glucose). Also, p85α (*p* = 0.030) and p110β (*p* = 0.003) expressions were higher than in the IEC incubated with 5.5 mM glucose.

#### 3.5.2. Jejunal Gluconeogenesis/Glycolysis

Regarding the IEC treated with 5.5 mM glucose (Figure 4), the incubation with a high dose of insulin (5.5 mM glucose + 100 nM insulin) produced an increase of GLUT1 (*p* = 0.033) expression, and a decrease of the glycolytic enzymes PKLR (*p* = 0.05) and HK1 (*p* = 0.01). The incubation with high levels of glucose (25 mM glucose) produced an increase of PEPCK (*p* = 0.035), and a decrease of GLUT1 (*p* = 0.023) and the glycolytic enzymes PKLR (*p* = 0.001) and HK1 (*p* = 0.002) when compared with IEC treated with 5.5 mM glucose. 

In the IEC incubated with a high dose of glucose and insulin (25 mM glucose + 100 nM insulin) (Figure 4), PEPCK expression (*p* = 0.035) was higher and GLUT1 (*p* = 0.028) and HK1 (*p* = 0.028) lower than in the IEC incubated with a high dose of insulin (5.5 mM glucose + 100 nM insulin). GLUT1 expression (*p* = 0.046) was lower than in the IEC incubated with a high dose of glucose (25 mM glucose). Also, PEPCK expression (*p* = 0.025) was higher and the glycolytic enzymes HK1 (*p* = 0.02) and PKLR (*p* = 0.015) lower than in the IEC incubated with 5.5 mM glucose.

### 3.6. Leptin Effects on the mRNA Expression Levels in Incubated IEC

**Figure 5:**
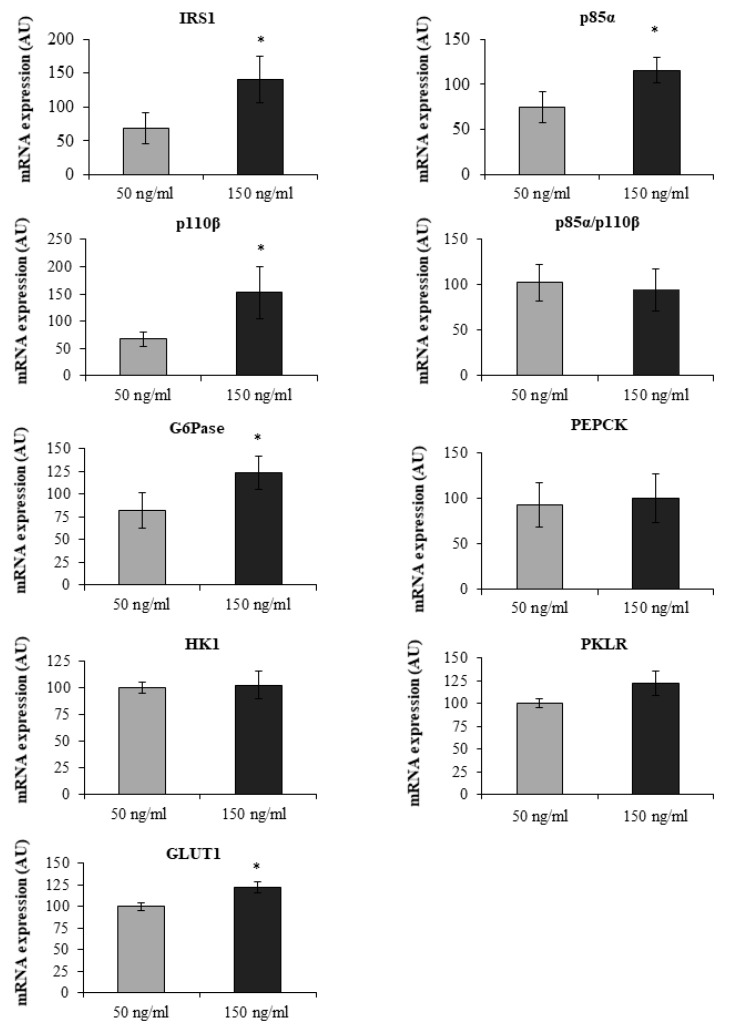
mRNA expression levels of studied genes in intestinal epithelial cells (IEC) incubations obtained from jejunum samples of MO-low-IR (*n* = 6). IEC were isolated and incubated as described in the Material and Methods section. IEC were incubated with 50 mg/mL leptin and 150 mg/mL leptin. The results were expressed as a percentage of 50 mg/mL leptin (100%). * Significant differences between 50 mg/mL and 150 mg/mL (*p* < 0.05). The results were given as the mean ± SEM. IRS1: insulin receptor substrate 1. p85α: phosphatidylinositol 3-kinase 85 kDa regulatory subunit alpha. p110β: phosphatidylinositol 3-kinase 110 kDa catalytic subunit beta. G6Pase: glucose-6 phosphatase. HK1: hexokinase 1. PKLR: L-type pyruvate kinase. GLUT1: solute carrier family 2 member 1 or glucose transporter 1. PEPCK: phosphoenolpyruvate carboxykinase.

As insulin and leptin can act through the PI3K pathway [19] and they are associated with IRS1, we checked whether leptin could modify the gene expression of IEC. At a high dose of leptin (150 ng/mL), IRS1, (*p* = 0.043), p85α (*p* = 0.043), p110β (*p* = 0.043), GLUT1 (*p* = 0.018) and G6Pase (*p* = 0.020) expressions were significantly higher than in IEC incubated with 50 ng/mL of leptin (Figure 5).

## 4. Discussion

The results of the study show that in the jejunum of MO-high-IR there is an increase of IRS1 and p110β and a decrease of the p85α/p110β ratio. Also, there is an increased expression in glycolytic (HK1 and PKLR) and gluconeogenic enzymes (PEPCK and G6Pase), and a decrease in phospho-Akt levels. This altered profile is slightly attenuated in MO-metf-T2DM. The IRS1, p85α/p110β ratio, HK1 and G6Pase expression are also associated with the changes in BMI and HOMA-IR after RYGB. The expression of most of the studied genes involved in the insulin signalling pathway in IEC is regulated by glucose + insulin and/or leptin.

One of the main finding of this study is the increase in IRS1 and the decrease in the p85α/p110β ratio in MO-high-IR. In human adipose tissue and skeletal muscle, a decrease in IRS1 levels has been described in a state of insulin resistance [27,28], although there are contradictory results [28]. In this regard, an increase in insulin resistance of intestinal enterocytes has been shown in morbidly obese subjects [29]. The authors suggest that intestinal insulin resistance may appear before serum insulin resistance, and may be independent of glycaemic status [29]. Despite this increase in IRS1, our data show a decrease in Akt phosphorylation, protein involved in the regulation of intracellular signalling of insulin. This would confirm an increase in insulin resistance in jejunum from those morbidly obese subjects with high systemic insulin resistance. This decrease in phospho-Akt is also found in the duodenum of insulin-resistant morbidly obese subjects [30]. On the other hand, we have found an increase in p110β and a decrease of p85α/p110β ratio. This agrees with a previous study showing p85α mRNA unchanged in primary human skeletal and adipose tissue of type 2 diabetics [31]. However, in the human intestine, there is no information about p85α and p110β mRNA expression levels. An impairment in the PI3K pathway is usually associated with glucose intolerance and insulin resistance [16,17]. An excess of p85 isoform over p110 isoform (increased p85α/p110β ratio) can compete with the p85α/p110β enzyme for binding to IRS. However, an imbalance of the p85α/p110β ratio could cause either increased or decreased PI3K activity [32], which could impair insulin signalling and produce insulin resistance [16,33].

Our data could also suggest an increase in the cycle gluconeogenesis/glycolysis in MO-high-IR. We have found an increased expression of genes involved in glycolysis (HK1 and PKLR) and gluconeogenesis (PEPCK and G6Pase expression and G6Pase activity). However, the decrease found in the expression of genes involved in gluconeogenesis/glycolysis in those subjects treated with metformin was not observed for G6Pase expression and activity. This suggests that the glucose-6-phosphate continues to turn into glucose, with a possible greater release to blood. Another important finding is that IRS1 and p110β expression decreases in MO-metf-T2DM. To our knowledge, there are no studies evaluating the effect of metformin on intestinal insulin signalling.

GLUT1 is involved in the insulin-independent glucose intake in the enterocyte. The increase of GLUT1 in MO-high-IR suggests that the enterocytes would have a higher glucose uptake at baseline in insulin-resistant state, slightly decreasing in those subjects in metformin treatment. This could be the result of the combination of its stimulation by leptin and insulin, as we found in the in vitro incubations and in other studies [34,35]. Also, this agrees with the increase found in gluconeogenesis/glycolysis. In subjects with high insulin resistance, there would be an increase of gluconeogenesis, with an increased G6Pase activity and glucose production, and a possible increase in glucose uptake due to GLUT1. This would produce an increase of glycolysis to compensate the increase of glucose level into cells. As a result, stimulation of HK1 and hexosamine pathway would occur [36]. It is known that hexosamines have a negative feedback effect on GLUT4, leading to insulin resistance [36].

The correlations found in this study suggest that IRS1, p85α and/or p110β expression may be regulated by molecules such as insulin and leptin. It is known that insulin and leptin share signalling pathways. Leptin may be involved in the regulation of glucose metabolism [37]. Our in vitro results suggest that leptin could be responsible for the increased IRS1 and p110β mRNA levels found in MO-high-IR. These subjects had also the highest leptin level. While the expression of hepatic IRS1 was decreased in diabetic mice, leptin treatment at physiological doses considerably restored the expression levels of IRS1 [38]. This effect of leptin on p110β, although not on IRS1, was also produced by insulin. Insulin produced an increase of p110β expression and mainly of p85α. As a consequence, an increase in p85α/p110β ratio was found. However, the regulation of p85α seems to be complex. Although leptin and/or high glucose + insulin levels produced in vitro an increase in p85α, its level was decreased in an insulin resistance state. Our results agree with a previous study showing that insulin enhances the transcription of p85α in human skeletal muscle cultures, but is blocked in the skeletal muscle of type 2 diabetic patients after a 3 h hyperinsulinaemic euglycaemic clamp [31]. It is possible that p85α may be regulated by factors different to those involved in the p110β regulation.

Our results also suggest that the cycle gluconeogenesis/glycolysis is regulated by insulin/leptin. High leptin levels increased G6Pase in IEC, which could contribute to the higher expression of gluconeogenic enzymes found in MO-high-IR. Interestingly, we have found a decrease in glycolytic enzymes HK1 and PKLR when IEC were incubated with high doses of glucose or insulin. However, we do not know the mechanism or the reason for the decrease of the expression of these enzymes. We could hypothesize that IECs in a state of non-insulin resistance might not metabolize a large amount of glucose under normal conditions so as not to increase hexosamine levels, since they may lead to insulin resistance [36]. So, the excess of glucose would be transported again outside the cells, as some studies suggest [39,40]. GLUTs in the basolateral membrane of IEC transport glucose from the cytoplasm, where glucose accumulates, generating a gradient that favours its transport outside, to the interstitium [39,40].

However, what are the clinical implications of our results? Abnormalities in intestinal insulin signalling could be present in several diseases, including obesity, diabetes and cancer and could contribute to/prevent the comorbidities associated with these diseases. We found that a high IRS1, HK1 and GLUT1 expression and a low p85α/p110β ratio are associated with the changes of BMI and HOMA-IR after RYGB. However, this relation between leptin, the PI3K signal pathway and insulin resistance can be involved in other pathologies, such as colorectal cancer. A meta-analysis reported a relationship between BMI and colorectal cancer [41]. Conversely, BMI reduction was suggested as reducing the risk of colon cancer development [42]. In this context, leptin and PI3K can be involved in the obesity–colorectal cancer association [43]: leptin activates PI3K in the colon cancer cells HCT-116 and could regulate the proliferation of colorectal carcinoma through the PI3K signalling pathway [44]. It is known that PI3K promotes carcinogenesis and metastasis of colon cancer.

This study has several limitations. We think that the studied proteins may be the most interesting ones involved in insulin signalling and glycolysis. However, other proteins are also involved, as those of the leptin pathway. We did not obtain enough tissue to measure all the protein and phosphorylation levels and the enzymatic activity of the studied genes. Also, it would have been interesting to introduce a control group of lean patients. However, we could not obtain jejunal biopsies from the same location of healthy non-obese patients.

## 5. Conclusions

Our results show that in the jejunum of MO-high-IR, the IRS1, p110β, HK1 and G6Pase expression, the p85α/p110β ratio and the level of Akt phosphorylation are altered. These molecules are involved in the regulation of the insulin resistance and glucose metabolism pathways. On the other hand, metformin could be slightly counterbalancing this situation. Moreover, these genes involved in insulin signalling are associated with the changes of HOMA-IR after RYGB. We also suggest that leptin could play an important role in the increased IRS1 and p110β mRNA levels found in MO-high-IR. Together, our results could imply a great involvement of jejunum in the regulation of serum glucose levels.

## Figures and Tables

**Table 1:** Anthropometric and biochemical variables of the morbidly obese subjects.

	MO-Low-IR	MO-High-IR	MO-Metf-T2DM
N (men/women)	15 (6/9)	15 (5/10)	15 (5/10)
Age (years)	40.3 ± 9.8	42.2 ± 5.1	44.2 ± 12.9
Weight (kg)	129.3 ± 16.8	159.3 ± 35.5 *	126.7 ± 12.8 ^1^
BMI (kg/m^2^)	46.8 ± 6.1	54.1 ± 8.0 *	47.9 ± 6.3
Waist (cm)	131.6 ± 13.6	151.7 ± 17.2 *	133.5 ± 10.6
Hip (cm)	146.4 ± 7.6	157.7 ± 13.1	139.2 ± 14.5 ^1^
Glucose (mg/dL)	88.3 ± 8.8	116.3 ± 50.9 ^#^	136.5 ± 51.6 ^b^
Insulin (µIU/mL)	13.1 ± 1.9	28.6 ± 9.1 ^†^	18.5 ± 7.6 ^a,1^
Cholesterol (mg/dL)	215.4 ± 62.2	209.3 ± 30.5	208.7 ± 20.5
Triglycerides (mg/dL)	106.5 ± 31.1	141.8 ± 49.3	186.5 ± 52.6 ^a,1^
HOMA-IR	2.83 ± 0.50	8.26 ± 3.09 ^†^	6.21 ± 2.10 ^c^
Leptin	49.2 ± 15.6	101.6 ± 49.1 *	57.9 ± 32.7

The results are given as the mean ± SD. BMI: Body mass index. HOMA-IR: homeostasis model assessment of insulin resistance index. Significant differences between MO-low-IR (low HOMA-IR value) and MO-high-IR (high HOMA-IR value) groups: * *p* < 0.05, ^#^ *p* < 0.01, ^†^ *p* < 0.001. Significant differences between MO-low-IR and MO-metf-T2DM groups: ^a^ *p* < 0.05, ^b^ *p* < 0.01, ^c^ *p* < 0.001. Significant differences between MO-high-IR and MO-metf-T2DM groups: ^1^ *p* < 0.05. MO-metf-T2DM: group with type 2 diabetes mellitus (T2DM) who were only receiving metformin treatment.

**Table 2:** Significant correlations found between mRNA expression levels with anthropometric and biochemical variables.

	Insulin *	HOMA-IR *	BMI	Waist *
IRS1	*r* = 0.487; *p* = 0.003	*r* = 0.443; *p* = 0.008	Ns	Ns
p110β	*r* = 0.370; *p* = 0.048	*r* = 0.379; *p* = 0.043	Ns	Ns
p85α/p110β	*r* = −0.558; *p* = 0.002	*r* = -0.628; *p* < 0.001	*r* = −0.476; *p* = 0.009	*r* = −0.687; *p* = 0.001
G6Pase	*r* = 0.461; *p* = 0.005	*r* = 0.424; *p* = 0.010	Ns	Ns
PEPCK	*r* = 0.347; *p* = 0.045	Ns	*r* = 0.416; *p* = 0.025	*r* = 0.446; *p* = 0.043
HK1	*r* = 0.380; *p* = 0.022	*r* = 0.526; *p* = 0.001	*r* = 0.360; *p* = 0.024	*r* = 0.479; *p* = 0.007

BMI: Body mass index. HOMA-IR: homeostasis model assessment of insulin resistance index. * Adjustments for BMI were performed in the correlation analyses. Ns: Not significant.

**Table 3:** Multiple lineal regression models for the mRNA expression of the genes studied.

	Dependent Variable	*r* ^2^	Significant Independent Variables
Model 1	*p*85α	0.212	BMI (B = −0.406; *p* = 0.046)
	*p*110β	0.091	Insulin (B = 0.376; *p* = 0.047)
	*p*85α/p110β	0.208	Insulin (B = −0.448; *p* = 0.013)
Model 2	IRS1	0.822	Insulin (B = 0.611; *p* = 0.009) Leptin (B = 0.514; *p* = 0.032)

Model 1: Independent variables: Serum glucose, insulin and body mass index (BMI). Model 2: Independent variables: Serum glucose, insulin, body mass index and leptin.

**Table 4:** Body mass index (BMI), insulin resistance (HOMA-IR) and their changes (Δ) after RYGB.

	Presurgery	1 Month after RYGB	3 Months after RYGB	6 Months after RYGB	12 Months after RYGB
BMI (kg/m^2^)	49.2 ± 7.5 ^a^	42.3 ± 5.7 ^b^	37.3 ± 4.8 ^c^	34.9 ± 4.8 ^d^	31.2 ± 3.2 ^e^
ΔBMI	-	12.9 ± 3.2 ^d^	21.9 ± 4.2 ^c^	28.8 ± 5.5 ^b^	35.2 ± 7.6 ^a^
HOMA-IR	5.9 ± 3.9 ^a^	3.4 ± 2.3 ^b^	2.3 ± 1.0 ^c^	1.6 ± 0.8 ^d^	1.4 ± 0.8 ^d^
ΔHOMA-IR	-	26.6 ± 29.5 ^d^	49.9 ± 23.8 ^c^	64.9 ± 17.4 ^b^	66.2 ± 16.8 ^a^

Different letters indicate significant differences between the means (*p* < 0.05).

**Table 5:** Significant associations found between baseline expression levels of the studied genes and the evolution of body mass index (BMI) and insulin resistance (HOMA-IR) after RYGB.

	Months after RYGB	IRS1	p85α/p110β
ΔBMI	1	*r* = 0.718; *p* < 0.001	NS
	3	*r* = 0.594; *p* = 0.003	*r* = −0.440; *p* = 0.028
	6	*r* = 0.490; *p* = 0.024	Ns
	12	Ns	Ns
ΔHOMA-IR *	1	Ns	*r* = −0.610; *p* = 0.004
	3	*r* = 0.641; *p* = 0.002	*r* = −0.685; *p* = 0.001
	6	*r* = 0.663; *p* = 0.004	*r* = −0.641; *p* = 0.002
	12	*r* = 0.454; *p* = 0.045	*r* = −0.513; *p* = 0.035

* Adjusted by baseline body mass index. NS: Not significant.
